# European Consumers’ Willingness to Pay for Red Meat Labelling Attributes

**DOI:** 10.3390/ani11020556

**Published:** 2021-02-20

**Authors:** Emilia Cubero Dudinskaya, Simona Naspetti, Georgios Arsenos, Emmanuelle Caramelle-Holtz, Terhi Latvala, Daniel Martin-Collado, Stefano Orsini, Emel Ozturk, Raffaele Zanoli

**Affiliations:** 1 Department of Agricultural, Food and Environmental Sciences (D3A), Università Politecnica delle Marche, Via Brecce Bianche, 60131 Ancona, Italy; ozturk@agrecon.univpm.it; 2 Department of Materials, Environmental Sciences and Urban Planning (SIMAU), Università Politecnica delle Marche, Via Brecce Bianche, 60131 Ancona, Italy; 3 Faculty of Veterinary Medicine, Aristotle University of Thessaloniki, P.O. Box 393, GR-54124 Thessaloniki, Greece; arsenosg@vet.auth.gr; 4 The French Livestock Institute/Institut de l’Elevage (IDELE), Campus INRA—Chemin de Borde Rouge, CEDEX, BP 42118-31321 Castanet Tolosan, France; Emmanuelle.Caramelle-Holtz@idele.fr; 5 Natural Resources Institute Finland (Luke), P.O. Box 2, FI-00791 Helsinki, Finland; terhi.latvala@luke.fi; 6 Animal Production and Health Unit, Agrifood Research and Technology Centre of Aragon (CITA), Gobierno de Aragón, Avenida Montañana 93, 050059 Zaragoza, Spain; dmartin@cita-aragon.es; 7 Organic Research Centre, Trent Lodge, Stroud Road, Cirencester, Gloucestershire GL7 6JN, UK; stefano.o@organicresearchcentre.com

**Keywords:** consumer preferences, discrete choice experiment (DCE), organic food and farming, country-of-origin, fat, Halal, carbon footprint, protein content, convenience

## Abstract

**Simple Summary:**

Given the decrease in red meat consumption in the last decade, it is crucial for red meat producers to understand consumer preferences. This international study analyses the European consumer preferences for red meat (beef, lamb and goat) in seven countries: Finland, France, Greece, Italy, Spain, Turkey and the United Kingdom. Through a survey with hypothetical choice situations (choice experiment), 2900 responses were collected. Advanced econometric models were estimated to identify the diversity of preferences among consumers at the country level. The results indicate substantial differences between the most relevant attributes for the average consumer in each country. Nevertheless, national origin and organic labels were highly valued in most countries.

**Abstract:**

Food consumption in Europe is changing. Red meat consumption has been steadily decreasing in the past decades. The rising interest of consumers for healthier and more sustainable meat products provides red meat producers with the opportunity to differentiate their offers by ecolabels, origin and health claims. This international study analyses the European consumer preferences for red meat (beef, lamb and goat) in seven countries: Finland, France, Greece, Italy, Spain, Turkey and the United Kingdom. Through a choice experiment, 2900 responses were collected. Mixed multinomial logit models were estimated to identify heterogeneous preferences among consumers at the country level. The results indicate substantial differences between the most relevant attributes for the average consumer, as well as their willingness to pay for them in each country. Nevertheless, national origin and organic labels were highly valued in most countries.

## 1. Introduction

Food production and consumption tendencies in Europe have shown significant changes in recent years, especially regarding meat consumption. While beef and lamb meat production have decreased since the beginning of the nineties, poultry and pig meat production have increased during the same period [1,2]. Concerns about health issues and environmental sustainability have driven away beef consumers [3,4,5,6]. Its peculiar taste, with consumers perceiving it as fatty, difficult to cook, and sold in family-sized portions, has negatively impacted lamb meat consumption. Nevertheless, when new lamb meat products were introduced for younger consumers (e.g., single portions), lamb meat sales increased [7]. Addressing specific consumer needs through product differentiation could help revitalise the European red meat market [8].

In Europe, the rising interest in societal benefits (e.g., sustainability, biodiversity) as well as the growing concerns on more “ethical” food production methods [9,10] provide red meat producers with the opportunity to compete through differentiation. However, societal benefits are usually embedded in credence cues that the consumers cannot evaluate before or even after consumption, given the lack of expertise or practical possibilities [11]. In this case, meat producers must find alternative ways to communicate such benefits. The use of ecolabels and specific claims represent tools to inform the concerned consumer about attributes of interest. However, even if a specific label or claim is perceived positively by consumers, willingness to pay for that attribute might vary, as consumer preferences and purchase intentions are heterogeneous between countries [12].

This study presents the results of an econometric analysis of consumer preferences and willingness to pay for health-related and ethical claims in the red meat European market. Previous studies have evaluated consumer preferences for either beef, lamb or goat meat individually and mainly focused on specific national contexts [8,13,14]. However, comprehensive trans-national research comparing preferences for all red meat types and their key attributes was still missing. This study aims to fill this gap through a discrete choice experiment (DCE) on six European countries and Turkey.

Based on a systematic literature review on Scopus and Web of Knowledge, 458 papers were identified using keywords related to consumption, meat, choice experiments/choice model or conjoint analysis/conjoint design. Only studies published in English in peer-reviewed journals were considered. Articles in which the main subject under study was not the consumer or the consumer’s perception of meat attributes were excluded. Additionally, the definition of meat was restricted to fresh meat, avoiding processed products (e.g., sausages, ham) and fish. Only studies using choice experiments or conjoint analysis were taken into account, retrieving 92 articles.

After analysing these articles, the most significant attributes for the consumer when selecting meat were identified as: origin [15,16,17], price [18,19], animal welfare [20,21,22], fat content [13,23,24], type and cut of meat [25,26,27], organic [28,29,30], carbon footprint [31,32,33], certifications such as Protected Geographical Indication (PGI) and Protected Denomination of Origin (PDO) [8,34,35], nutritional/health claims [36,37,38] and seasoning/natural presentations [39,40].

According to previous studies, consumers tend to prefer meat of local or national origin [16,41]. However, other studies found that attributes related to quality and traceability are often more important than the meat’s origin [19]. In Europe, origin certifications often embed a quality assurance scheme, for example, PDO and PGI in the European Union [42]. The PGI label emphasises that the quality, reputation or other characteristics of the product itself is primarily attributable to its geographic origin. The Protected Designation of Origin (PDO) label identifies a product whose characteristics or quality are originated by having every part of the production, processing and preparation process taking place in a particular geographical location [43,44]. Nevertheless, studies involving these quality schemes are limited [45,46].

The organic label is also perceived as a proxy for product quality. In the European Union, the mandatory organic logo (Euroleaf) guarantees that the products have been produced following the best environmental practices, a high level of biodiversity, the preservation of natural resources, the application of high animal welfare standards, and a production method using natural substances and processes [47]. Although organic products have gained high interest by consumers [28], perceiving them as healthier, safer and with better organoleptic qualities [29,48,49,50,51], there are still conflicting results regarding consumers’ willingness to pay for them [27,39,52,53]. The carbon footprint label also shows consumers the commitment to environmental sustainability that the producers have [54]. However, previous research reported contradictory results on the importance that consumers give to this label when making their choices [9,33,55].

Although the average consumer is concerned about animal welfare [28,56,57], previous studies showed heterogeneous preferences [32,37]. According to de Jonge and van Triip [21], this variety of results is a product of the complexity of the animal welfare concept, which is highly dependent on consumer perception, their inferences on the diverse animal welfare levels, as well as how the message is communicated. In this study, given the difficulty of communicating animal welfare claims beyond the EU-wide animal welfare standards, animal welfare was not included in the attributes. Instead, the Halal certification label was included. Though Islam does not tolerate animal abuse, most halal certification does not incorporate specific animal welfare requirements, while pre-slaughter stunning is still a contentious issue [58]. Islamic rules require the animals to be alive, healthy, fed and watered before the slaughtering. The animal must be killed incurring the least pain possible, and the carcass must be drained of blood [59]. Some European Union countries (i.e., Belgium and Denmark) require that all animals be previously stunned before Halal slaughtering, while a recent EU Court ruling has established that all organic meat needs to be stunned, including that bearing the Halal label [60,61]. Research on reversible pre-slaughter stunning is still ongoing to improve animal welfare during slaughter yet still meet the spiritual requirements for Halal [58]. Previous research also identified that many consumers, including non-Muslims, perceive the Halal label as a credence quality attribute [62,63], and this is a growing trend [64]. There is not much evidence on consumer perception of Halal certification in Europe, though one study showed that some non-Muslims question animal welfare issues in relation to the Halal certification in the UK [65]. However, a recent EU-funded study concluded that since “there is little expressed use of animal welfare as a purchase criterion, little understanding of the slaughter process and an inability to distinguish between different methods of stun, providing additional information on the different methods used would not appear to aid a consumer decision” [66].

There is also a wide variety of nutritional and health labels used for diverse purposes. As red meat remains the most important dietary source of protein [67], a “high protein content” claim was selected for the present study. There is little previous literature reporting studies on consumers’ preferences of protein content in meat products [36,68], showing significant differences in cross-cultural settings. Moreover, there is a growing trend to use ready to cook/eat foods [69] and to look for healthier products [56] with less fat. However, the previous literature does not offer clear and homogeneous results in both cases, as consumers present opposite views regarding these attributes’ desirability, according to their culture and depending on the meat cut [38].

Given the variability of results from prior studies, all the previously mentioned attributes were included in this research. In addition, to account for cross-cultural differences, the willingness to pay (WTP) for each attribute was estimated at country level. Previous studies have established that product differences can be product differentiators only if they create valued benefits that consumers are willing to pay for [70]. Understanding the value that consumers give to each meat attribute and their WTP will provide essential information to producers, allowing them to identify which labels and quality claims consumers are willing to pay for.

The present paper is organised as follows. In the next section, there is a description of the methods applied, followed by the presentation of the results for each country. Then, the results are discussed, and conclusions are presented in the final section.

## 2. Materials and Methods

### 2.1. The Discrete Choice Model

A DCE is a survey-based methodology widely used and well established for modelling consumers’ preferences [71,72]. The method simulates a trading market with different attribute combinations. “Different levels of item attributes are combined, and these combinations are configured into a choice set according to the unbiased and efficient principles of statistical estimation” [73]. In each choice set, respondents select the option they like the most, after comparing the given alternatives. All alternatives in a choice set are described by the same attributes, and each of these attributes can take one level from a set of possible levels (e.g., the presence or absence of the organic label) [74]. Moreover, by including price/cost as one of the attributes of the good, the willingness to pay of a respondent for a specific attribute can also be calculated.

The theoretical model is based on the Lancastrian consumer theory [75] and the random utility model (RUM) framework [76], in which the utilities of different goods can be broken down into separate utilities for their attributes. Faced with a set of *J* available alternatives, rational decision-makers would select the alternative with the highest utility to them. This utility is known to the decision-maker, but not to the researcher [77]. 

Since there are aspects of utility that are unknown to the researcher, the total utility for the alternative j for the respondent i is decomposed as:

U_ij_ = V_ij_ + ε_ij_ (1)

where the researcher can only observe V_ij_, the deterministic part of the utility. The remaining part of the utility (ε_ij_) is unobservable for the researcher and treated as random. The systematic component (V_ij_) can be approximated by a linear function of observed attributes in the vector X_ij_ and the utility parameters of each attribute collected in the vector β:

V_ij_ = βX_ij_ (2)

Assuming heterogeneous individual preferences across respondents [78], the discrete choice model may be specified as a mixed multinomial logit (MMNL) model that allows the unobserved, random part of the utility to follow any distribution [79].

Thus, the utility of individual i from alternative *j* is specified as [77]:

U_ij_ = β′_i_ X_ij_ + ε_ij_ (3)

where β’_i_, is a vector of unobserved coefficients that varies between individuals but not over alternatives, X_ij_ is a vector of observed variables that relate to each alternative *j* and respondent i, and ε_ij_ is a random term that is i.i.d. extreme value over individuals and alternatives. For the present research, the linear specification of the utility for an individual i for the alternative j is:

U_ij_ = α_ij_ + β_price_ Price + β_halal_ Halal + β_national origin_ National origin + β_eu origin_ EU origin + β_PGI_ PGI + β_organic_ Organic + β_carbon_ Carbon + β_Low fat_ Low fat + β_protein_ Protein + β_format_ Format + ε_ij_ (4)

The random parameters β’ (except the price) were assumed to be normally distributed to allow both positive and negative preferences for each attribute. A negative lognormal distribution was assumed for the price parameter to attain better behavioural fit to microeconomic theory. Including the monetary cost in the choice experiment offers wide information on the trade-offs that consumers make among the benefits provided by the different alternatives (with diverse attributes) and their price, allowing the estimation of the WTP for each attribute. 

The utility was specified directly in the WTP space [80] to obtain better results and reduce the range of behavioural implausibility [81]:

U_ij_ = α_ij_ − λ_i_ p_ij_ + (λ_i_ γ_i_)’ x_ij_ + ε_ij_ (5)

where λ_i_ = (β_i price_/μ_i_). β_i price_ and μ_i_ are, respectively, an individual-specific coefficient for price and an individual-specific scale parameter; while γ_i_ = (c_i_/λ_i_), where c_i_ = (β_i_/μ_i_). 

Estimating an MMNL model directly in the WTP-space offers the direct advantage of obtaining parameters and estimated standard errors that can be immediately interpreted as marginal WTP values [80,82,83]. This means that the estimated results are already presented in the currency used to make the choice experiment, simplifying the interpretation of the results.

### 2.2. Product and Attributes Selection

A labelled DCE was developed, including four labelled red meat alternatives (lamb leg, lamb chops, goat chops, T-bone steak) and a no-choice option. The type of meat and cuts were based on the most consumed cuts of lamb and goat meat in the countries investigated, as they often present less variety than beef. As the most consumed lamb and goat cuts were chops and legs (for lamb), a T-bone steak was considered as the most comparable beef cut, to avoid bias for more convenient cuts or with no presence of bones.

Based on the literature review results and a qualitative study [84], nine credence attributes were selected (Table 1). Given the high number of attributes, the number of levels for each attribute was kept low to avoid excessive participants’ cognitive burden [85].

**Figure 1:**
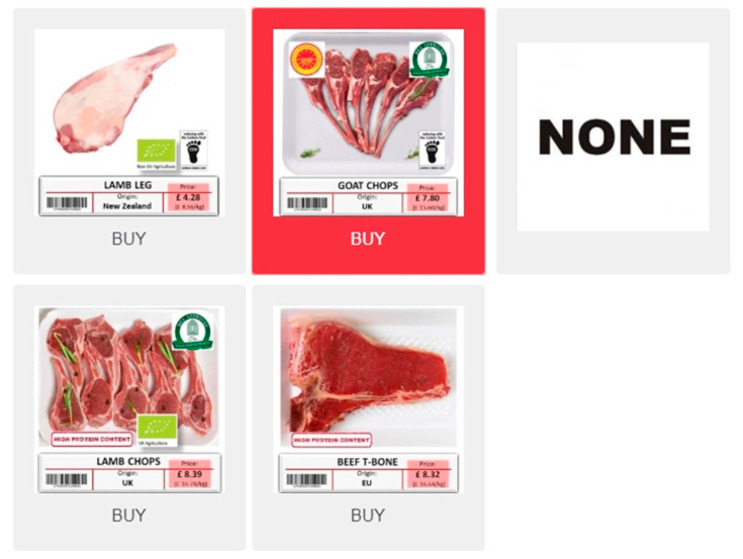
Example of a choice set.

All alternatives were presented to the respondent simultaneously with all their attributes (Figure 1). The product name, origin and price were introduced through text in each alternative, while the other attributes were presented graphically either using labels or by modifying the original image (see below an explanation for each attribute). To avoid any biases, only the attributes under study were modified (e.g., fat), keeping all the other characteristics (e.g., colour, size) unchanged between the images of the same product. The base price was calculated as the average price for each meat type and cut in each country. Then, it was pivoted in three levels, with variations of ±30%. The price was expressed in local currencies.

The origin attribute was set to three levels: national, EU and New Zealand, as most lamb meat imports to the EU come from this country [86]. New Zealand was set as the reference category for differences in utility in the choice model. As a consequence, the estimated willingness to pay (WTP) for both national and EU origin needs to be interpreted as a premium price above the New Zealand origin. 

The PGI and PDO labels were presented through their official labels in the primary language of each country. The choice to choose either PGI or PDO was based on the presence/absence or predominance of each certification scheme for lamb meat in each country. In Finland, there exists only one PDO for red meat. In Turkey, being outside the EU, PGI and PDO regulations do not apply. Therefore, in both countries, the PGI and PDO labels for national meat were omitted as there are no certifications available in these countries. 

The organic label was also adapted by country. Turkish respondents were presented with their local logo, while, for the European countries, the mandatory EU organic logo was used. The Halal certification, the carbon footprint and the “high protein content” labels were also adapted to each country. With respect to the carbon footprint attribute, the Carbon Trust label was used, which has gained popularity in the UK in recent years. The label was presented in the primary language of each country. In the case of the “high protein content” claim, a simple label of red letters on a white background was created for the present research, as there was no homogeneous label used across the countries under study.

**Figure 2:**
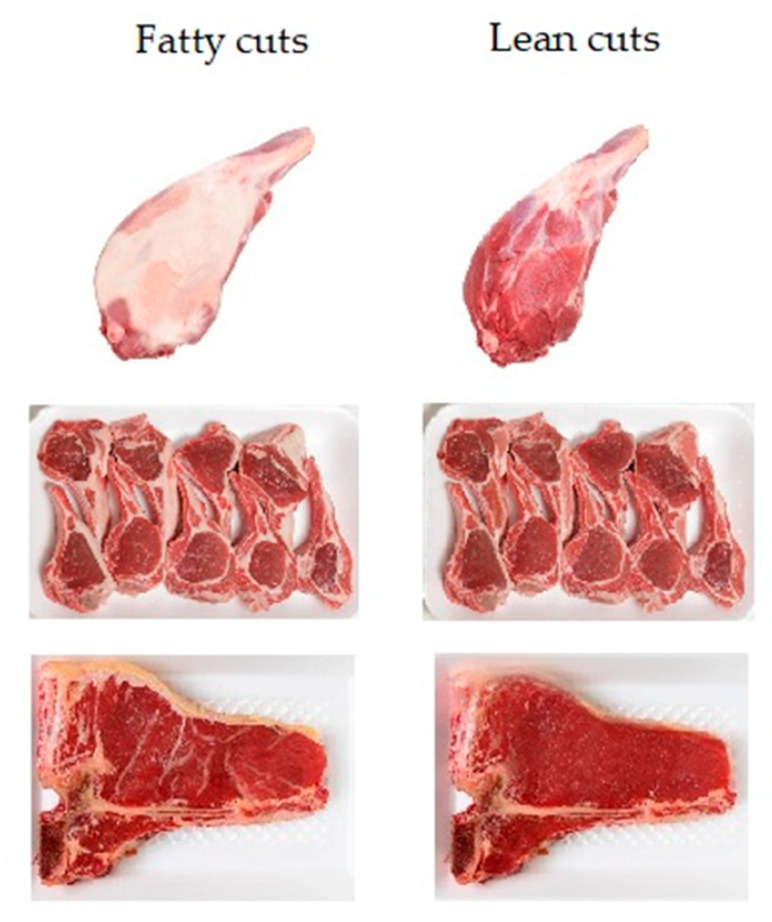
Examples of fat content presented in red meat cuts.

The attributes fat content and format (convenience) were introduced to the respondents by modifying the alternative’s image. For example, for fat content, the base image of each cut was modified using image-editing software by adding or removing the presence of visual fat, as shown in Figure 2. No additional claims (or labels) referring to the fat content or format were added in any way. The objective was to keep it closer to reality, as people in the supermarket usually see the meat cut and the visual presence of fat (as a proxy to healthiness and meat quality) [23,87,88] when making their choice. In the case of format, the image was modified by adding additional ingredients (e.g., rosemary, pepper) to feature a ready-to-cook product.

### 2.3. Data Collection and Analysis 

The data were simultaneously collected through an online survey from March to May 2019 in seven countries: Finland (FI), France (FR), Greece (GR), Italy (IT), Spain (ES), Turkey (TR) and the United Kingdom (UK). The respondents were selected by a third party (Qualtrics) using a quota sampling approach to achieve between-country comparability [89,90]. The quotas were established according to age and occupation by gender [91]. Only red meat consumers (at least once a year) were sampled. Respondents were between 18 and 64 years old, fully or partially responsible for the grocery shopping in their household and did not work or had a close relative working in the meat or catering industry. Two thousand nine hundred responses were collected. After removing incomplete or low-quality surveys (e.g., speeders, line-responses), 2866 usable responses were left (approximately 400 per country—see Table 2).

The questionnaire was developed in English. Then, it was translated and back-translated to the primary language of each country [92,93]. Active collaboration and feedback from international researchers allowed cross-country conceptual, functional, and category equivalence [94,95]. The questionnaire included consumer sociodemographic and geographic data, as well as a hypothetical discrete choice experiment (DCE). It was pilot tested in each country before launch.

### 2.4. DCE Design and Estimation

A fractional D-efficient design with priors consisting of twenty-four choice sets in two blocks was generated (D-error = 0.256, A-error = 0.754) using the Ngene software [96]. In contrast to orthogonal designs, efficient designs do not merely try to minimize the correlation in the data for estimation purposes, but aim to result in data that generate parameter estimates with as small as possible standard errors. Each respondent was presented with twelve choice sets of five alternatives. This means that each respondent saw twelve different combinations of the five alternatives (lamb leg, lamb chops, goat chops, T-bone steak and a no-choice option) with different attribute levels (e.g., different prices, origins) defined according to the design. All choice sets and alternatives were presented randomly to avoid order-effect [97]. A “cheap talk” was introduced to respondents before answering the DCE, aiming to reduce the hypothetical bias [98,99]. Participants were introduced to all labels, their meanings and definitions [100]. They were also shown an example of a choice set. Respondents had unlimited time to answer each choice set [101]. A total of 34,392 choices were collected.

The data were analysed using the APOLLO package in R [102]. For each country, an MMNL model in WTP-space was estimated based on the preference-space MMNL model’s priors. The log-likelihood was attained by Monte-Carlo simulation-based integration using Halton draws with 1000 replications. A scaling factor was used to ease convergence in the WTP-space.

## 3. Results

The respondents’ sociodemographic characteristics and meat consumption frequency are presented in Table 3. In all countries, most consumers declare to consume beef at least once a month. However, the percentage of consumers that consume lamb or goat meat with the same frequency varies in each nation. Turkey is the country with higher lamb and goat meat consumption, followed by the United Kingdom and Spain. Finland is the country with lower consumption frequency for both lamb and goat meat. 

The consumers’ choices regarding the meat cut also varied between countries (Table 4). Beef T-bone was the preferred choice in Finland. Beef T-bone was the preferred cut in Greece too, though it was a close race with lamb chops; on the contrary, in Italy, the lamb chops were the preferred cut, closely followed by beef T-bone. In Spain, Turkey and the UK, there was a clear preference for the lamb chops over other meat types. Only in France, the lamb leg was the preferred cut. In all countries, most consumers preferred to choose a meat alternative than selecting none.

### Willingness to Pay for Each Country

The results of the MMNL in WTP-space are presented in Table 5. National origin and the organic label are the only statistically significant and positive attributes for all countries. The attributes EU origin, PGI/PDO, carbon footprint and low fat had positive coefficients when significant, while the attributes ready to cook and high protein content presented negative coefficients when significant. The Halal attribute was statistically significant and positively valued only in Turkey, while in the other countries where it was significant, it was perceived negatively. However, it is worth mentioning that the statistical significance of the standard deviation of the Halal attribute is relatively high in several countries, implying a high heterogeneity of taste for this attribute within those countries.

Consumers from all countries presented the highest WTP for the meat of national origin if compared with New Zealand or EU origin. French consumers demonstrated the highest WTP (EUR) for national or EU origin, while the UK had the lowest WTP (EUR 0.47) for national meat. In general, meat consumers were willing to pay more for the geographical origin of the meat than for certification labels linked to the origin of the meat (PGO/PGI). The PGO/PGI certification was significant and positive only in Greece, Italy, Turkey and the UK.

In most countries, the second preferred attribute was the organic label (besides in the UK, where the WTP for organic meat was higher than for the national origin). France presented the highest WTP for the organic label, followed by Greece, Finland and Italy. Another certification label included in the meat attributes was the carbon footprint, which was significant and positive in France, Greece, Spain and Turkey. However, besides Spain, most countries presented a higher WTP for other attributes (e.g., organic) than for the carbon footprint.

While the low-fat attribute was significant and positively valued only by French and Italian consumers, the high protein content was significant and negatively valued by Finnish, Greek and Turkish consumers. Moreover, the ready to cook format was also perceived negatively by France, Greece and Spain. However, the statistical significance of the standard deviations for this attribute might suggest heterogeneous preferences within the countries.

## 4. Discussion

Altogether, the results showed heterogeneity in consumer preferences and their WTP, both within and between countries. However, the national origin was always among the most valued attributes in all countries, in line with previous studies showing a higher preference for local and national products [41,52,103]. The EU origin was also valued positively and preferred over New Zealand imports, except for the UK. There was no significant difference in their WTP for meat coming from the EU and New Zealand for British consumers. New Zealand had a well-established position in the UK as a red meat supplier, at least before Brexit [104], which results in a quite accepted alternative to local lamb and, partly, beef.

The results were quite different when geographical quality assurance schemes were taken into consideration instead of geographical origin. The implementation of PDO and PGI labels had significant and positive influence only in Greece, Italy, Turkey and the UK. However, in all four countries, the PDO/PGI certification was always among the top three preferred attributes. Nevertheless, it always presented a lower WTP than the national origin. Red meat consumers were more inclined to pay more for the meat’s geographical origin than for the quality cue linked to the production process being explicitly developed in a particular geographical area. 

Organic products were also highly valued in all countries. The organic label was the second most important attribute for most countries, matching previous studies’ results [51,105]. However, the WTP for organic meat varied from one country to another. In this case, France, followed by Greece, presented the highest WTP for organic meat, making both countries the most attractive market for organic meat products. On the other hand, Spain, Turkey and the UK had the lowest WTP for organic meat, but with a high and significant standard deviation. The reported results imply a high heterogeneity of preferences in these latter countries regarding the organic attribute, confirming Koistinen et al.’s [9] results. However, such a variety of preferences also implies that there might be space for premium niche markets interested in organic meat. Further research is needed to identify possible organic segments within each of these countries and their willingness to pay for organic products. 

The carbon footprint label was also positively valued by consumers in most countries (France, Greece, Spain and Turkey). Nevertheless, the WTP for this attribute was lower than other attributes, matching results from previous studies [33,56]. This result is not surprising, as currently the label is used mainly in the UK, but for some other food products (e.g., cow’s milk). As the label has not been adopted for meat, consumers are not used to it. Additionally, Mandolesi et al. [84] suggested that most lamb/goat consumers believe that the production systems for sheep/goat meat are less intensive, while more environmentally friendly than those implemented in the beef industry. Such perception might bias consumers to perceive the carbon footprint label as not highly informative. Previous research [52,55] supports this idea as the carbon footprint label was ineffective in influencing consumer behaviour.

Although previous research has identified Halal as a quality attribute even for non-Muslim consumers [62], the results of the current study showed little relevance of the label in the investigated countries. Except for consumers in Turkey and Greece (where the Halal attribute was not significant), consumers in the other countries negatively perceive the Halal label. However, the standard deviation estimate for Halal is significant and relatively high for all countries, meaning that there is a high heterogeneity of preferences within countries. As the survey sample was not representative of religion (the Muslim population in each country is frequently under-represented), these results might imply the possibility of niche markets within each country. Further research is required to determine the existence of non-Muslim consumer segments interested in the Halal attribute and their WTP for that attribute.

The lack of visible fat was appreciated only among French and Italian consumers, while the other countries were indifferent to this attribute. Fat content in meat is frequently used by consumers as a health indicator [23], leading them to choose leaner meat cuts, i.e., with less visible fat content [9,52,106]. Scozzafava et al. [34] concluded that Italian consumers presented a lower price sensitivity for marble steak only when the meat cut was bought for special occasions and not for everyday consumption. As respondents in this study were requested to make their choices thinking of an ordinary day, the results reported above do not disconfirm their findings. 

Different consumer knowledge may also explain the heterogeneity of preference on visible fat between countries. Grunert [107] identified a dysfunctional preference of consumers towards the fat content of meat, as consumers believed that a certain degree of marbling detracted juiciness and taste from their steak, when it is clearly the opposite. Baba et al. [13] concluded that the fat content is a relevant attribute mainly for uneducated consumers, who usually consider marbling and fat a negative attribute. The number of consumers who appreciate the role of marbling in meat tenderness and juiciness is often as high as those who fear the cholesterol content. In this study, the standard deviation for fat content is significant for all countries. Therefore, consumers’ preferences about fat are heterogenous within each country, meaning there is no consensus on the role of fat in meat. In most countries except France and Italy, marbling is, on average, appreciated. However, this is not so for all consumers, and even in France and Italy some consumers appreciate fatter meat.

The high protein attribute was statistically significant and negative only in Finland, Greece and Turkey. Such a result implies that consumers were either indifferent or rejected the attribute. Although consumers tend to value additional nutrients positively [108], this is not the case for the protein content. However, Van Wezemael et al. [36] highlight the importance of how the claim is written. Teratanavat and Hooker [53] support this thesis by warning that claims referring to naturally-sourced nutrients are preferred over fortification or artificial enrichment. In this study, the protein claim did not explicitly specify the origin of the proteins; consumers might have perceived them as a fortification. Moreover, consumers may require additional information on the role of proteins for human health to appreciate such a claim. Further research is needed on the role and preference of proteins in meat products to understand how these claims are perceived and interpreted by the respondents.

The ready-to-cook attribute was significant and negative only in France, Greece, and Spain, implying that consumers from these countries prefer fresh products without any additional spices or preparations. However, in all nations, the attribute presented a significant standard deviation, which implies a high heterogeneity of preferences within countries. Such results suggest that there is a variety of different segments in each country, in which some respondents might appreciate the “ready-to-cook” preparations. Such findings match with the results from Pouta et al. [38], which concluded that consumers presented opposite views regarding the desirability of this attribute [38]. Further research is needed to identify the variety of preferences within countries. 

## 5. Conclusions

The present study looks at analysing the European meat consumer preferences and willingness to pay for health and “ethical” labels in beef, lamb and goat meat. Eight attributes were analysed through a hypothetical discrete choice experiment: national origin, EU origin, Halal meat, organic, carbon footprint, protein content, fat content, and ready-to-cook format. The willingness to pay was estimated for each significant attribute. The results show that there are significant differences between countries in terms of preferences and their willingness to pay for diverse attributes. However, national origin and organic are always the most preferred attributes. 

The results from this study have to be carefully interpreted as there are also some limitations. On the one hand, the study is not representative of the Muslim population in each of the countries, so there is not enough information to conclude how Muslim consumers would react to the Halal labels and trust them. Moreover, the study was limited to red meat eaters, so the results can only be generalized to red meat consumers. 

## Figures and Tables

**Table 1:** Attribute levels.

Attributes	Levels Considered
Price	Average price (country-specific) −30% +30%
Origin	National EU New Zealand (reference)
PGI/PDO	PGI/PDO No label (reference)
Organic label	Organic label No label (reference)
Halal label	Halal label No label (reference)
Carbon footprint label	Low carbon footprint label No label (reference)
Protein content label	High protein content label No label (reference)
Fat content	Low-fat Fatty (reference)
Format (convenience)	Ready-to-cook Plain (reference)

**Table 2:** Collected responses per country.

Country	Total Responses	Valid Responses
Finland	417	413
France	416	414
Greece	403	400
Italy	419	417
Spain	420	417
Turkey	405	391
UK	420	414
**Total**	**2900**	**2866**

**Table 3:** Sociodemographic characteristics of the sample in percentages by country.

Sociodemographic Variables	FI	FR	GR	IT	ES	TR	UK
Gender							
Female	49.4	50.7	48.5	49.6	50.4	49.1	50.0
Male	50.6	49.3	51.5	50.4	49.6	50.9	50.0
Age group							
18–24 years old	14.0	13.3	12.5	11.3	11.3	19.2	14.3
25–34 years old	21.3	20.8	19.5	18.2	19.2	28.1	21.5
35–44 years old	21.3	22.5	25.2	23.5	26.4	23.3	21.5
45–54 years old	22.3	23.4	23.8	26.4	23.5	16.4	23.4
55–64 years old	21.1	20.0	19.0	20.6	19.6	13.0	19.3
Occupation							
Employed	66.6	68.1	56.5	61.6	63.8	56.3	73.7
Unemployed	10.4	8.5	18.5	11.3	17.7	5.4	3.4
Inactive (retired + students)	23.0	23.4	25.0	27.1	18.5	38.3	22.9
Regular red meat consumers (at least once a month)							
Beef	77.2	87.2	76.5	89.9	82.3	76.2	79.0
Lamb	12.8	37.9	25.0	33.6	45.8	61.4	51.2
Goat	7.0	7.7	21.8	18.0	21.6	24.6	14.0
Total number of respondents	413	414	400	417	417	391	414

**Table 4:** Percentages of selected cuts in the discrete choice experiment (DCE) per country.

Cuts Selected in the DCE	FI	FR	GR	IT	ES	TR	UK
Beef T-bone	31	20	24	27	24	24	15
Goat chops	11	8	16	12	11	18	10
Lamb chops	18	21	23	29	29	29	30
Lamb leg	17	29	19	18	20	18	26
None	23	22	18	14	16	11	19
Total respondents	413	414	400	417	417	391	414

**Table 5:** Estimated willingness to pay (WTP) for all countries in local coin.

Estimates	Countries						
	FI (EUR)	FR (EUR)	GR (EUR)	IT (EUR)	ES (EUR)	TR (TRY)	UK (GBP)
LL ^a^	−6587.84	−6864.38	−6984.07	−7018.80	−7071.07	−6696.65	−6858.30
BIC ^b^	13,379.88	13,933.02	14,171.57	14,242.03	14,346.58	13,596.18	13,920.87
Adj. Rho-square	0.1711	0.1385	0.0928	0.1255	0.119	0.11	0.1392
Mean estimates (normal distribution)							
Halal	−0.693 (0.011)	−2.041 (0.000)	0.183 (0.493)	−0.542 (0.008)	−1.335 (0.000)	13.230 (0.000)	−0.713 (0.001)
National origin	2.277 (0.000)	3.737 (0.000)	2.299 (0.000)	3.052 (0.000)	2.584 (0.000)	11.070 (0.000)	0.433 (0.038)
EU origin	0.636 (0.022)	1.695 (0.000)	0.082 (0.783)	0.557 (0.018)	1.068 (0.002)	−0.993 (0.575)	0.143 (0.449)
PGI/PDO	0.035 (0.895)	0.357 (0.138)	0.973 (0.000)	0.815 (0.000)	0.472 (0.058)	6.857 (0.000)	0.302 (0.032)
Carbon footprint	0.330 (0.056)	0.495 (0.015)	0.412 (0.027)	−0.032 (0.827)	0.516 (0.022)	3.853 (0.001)	0.047 (0.681)
Organic	0.839 (0.000)	2.058 (0.000)	1.265 (0.000)	0.657 (0.000)	0.463 (0.036)	4.458 (0.000)	0.491 (0.004)
Low fat	0.330 (0.102)	1.134 (0.000)	0.181 (0.245)	0.554 (0.002)	0.357 (0.069)	0.242 (0.856)	0.137 (0.310)
High protein	−0.332 (0.049)	−0.147 (0.496)	−0.417 (0.011)	0.183 (0.257)	−0.150 (0.405)	−3.048 (0.001)	−0.136 (0.260)
Ready to cook	0.310 (0.101)	−0.705 (0.043)	−0.816 (0.000)	−0.200 (0.287)	−1.300 (0.000)	−1.646 (0.222)	−0.285 (0.097)
Standard deviations estimates (normal distribution)							
Halal	2.634 (0.000)	6.167 (0.000)	2.746 (0.000)	1.920 (0.000)	3.802 (0.000)	20.804 (0.000)	2.613 (0.000)
National origin	3.350 (0.000)	4.050 (0.000)	3.296 (0.000)	3.561 (0.000)	3.545 (0.000)	21.133 (0.000)	0.231 (0.826)
EU origin	1.105 (0.029)	1.421 (0.002)	1.803 (0.000)	0.185 (0.013)	2.273 (0.001)	11.164 (0.000)	0.009 (0.981)
PGI/PDO	0.407 (0.337)	0.361 (0.594)	0.893 (0.020)	0.667 (0.000)	0.784 (0.194)	5.862 (0.007)	0.514 (0.220)
Carbon footprint	0.250 (0.755)	1.180 (0.000)	1.536 (0.000)	0.768 (0.015)	1.495 (0.000)	8.220 (0.000)	0.388 (0.047)
Organic	2.101 (0.000)	3.065 (0.000)	1.731 (0.000)	0.987 (0.000)	2.065 (0.000)	6.354 (0.000)	1.449 (0.000)
Low fat	1.562 (0.000)	1.322 (0.000)	1.084 (0.000)	1.264 (0.000)	1.363 (0.000)	4.309 (0.084)	1.019 (0.000)
High protein	0.358 (0.101)	0.914 (0.088)	1.015 (0.019)	-0.083 (0.550)	0.545 (0.074)	0.979 (0.569)	0.194 (0.361)
Ready to cook	1.846 (0.000)	3.302 (0.000)	1.752 (0.000)	2.004 (0.000)	4.043 (0.000)	3.431 (0.001)	1.879 (0.000)

Numbers in parentheses are robust p-values. ^a^ LL: Value of Log Likelihood function ^b^ BIC: Bayesian information criterion.

## Data Availability

The datasets are available at: Zanoli, Raffaele (2021), “iSAGE red meat attributes choice experiment”, Mendeley Data, V1, doi:10.17632/psrybh3ksr.1. http://dx.doi.org/10.17632/psrybh3ksr.1
